# Quantum annealing feature selection on light-weight medical image datasets

**DOI:** 10.1038/s41598-025-14611-x

**Published:** 2025-08-07

**Authors:** Merlin A. Nau, Luca A. Nutricati, Bruno Camino, Paul A. Warburton, Andreas K. Maier

**Affiliations:** 1 Pattern Recognition Lab, Friedrich-Alexander-Universität Erlangen-Nürnberg, 91052 Erlangen, Germany; 2 London Centre of Nanotechnology, University College London, London, WC1H 0AH UK; 3 Rudolf Peierls Centre for Theoretical Physics, University of Oxford, Oxford, OX1 3PU UK; 4 Department of Chemistry, University College London, London, WC1H 0AJ UK; 5 Department of Electronic and Electrical Engineering, University College London, London, WC1E 7JE UK

**Keywords:** Medical imaging, Machine learning, Quantum computing, Quantum annealing, Image reconstruction, Medical imaging, Computational science

## Abstract

We investigate the use of quantum computing algorithms on real quantum hardware to tackle the computationally intensive task of feature selection for light-weight medical image datasets. Feature selection is often formulated as a *k* of *n* selection problem, where the complexity grows binomially with increasing *k* and *n*. Quantum computers, particularly quantum annealers, are well-suited for such problems, which may offer advantages under certain problem formulations. We present a method to solve larger feature selection instances than previously demonstrated on commercial quantum annealers. Our approach combines a linear Ising penalty mechanism with subsampling and thresholding techniques to enhance scalability. The method is tested in a toy problem where feature selection identifies pixel masks used to reconstruct small-scale medical images. We compare our approach against a range of feature selection strategies, including randomized baselines, classical supervised and unsupervised methods, combinatorial optimization via classical and quantum solvers, and learning-based feature representations. The results indicate that quantum annealing-based feature selection is effective for this simplified use case, demonstrating its potential in high-dimensional optimization tasks. However, its applicability to broader, real-world problems remains uncertain, given the current limitations of quantum computing hardware. While learned feature representations such as autoencoders achieve superior reconstruction performance, they do not offer the same level of interpretability or direct control over input feature selection as our approach.

## Introduction

Medical imaging plays a crucial role in modern clinical practice, providing essential insights for diagnosis, treatment planning, and monitoring. However, the increasing complexity and volume of imaging data present significant challenges for manual analysis. Machine learning (ML), particularly deep learning (DL), has emerged as a transformative tool to automate tasks such as disease classification, segmentation, and outcome prediction^1,2^. By learning complex representations from large datasets, DL methods have substantially improved diagnostic performance. Nevertheless, most ML models, especially those involving high-dimensional inputs like images, scale computationally with both the number of samples and the number of features, leading to high resource demands.

**Fig. 1:**
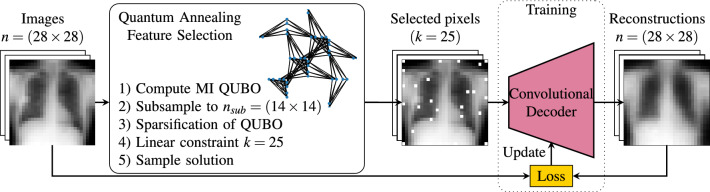
Illustration of the feature selection process using quantum annealing to extract pixels and train a convolutional decoder for reconstruction. The image dataset is used to compute mutual information (MI) between pixels and class labels (for importance) and between pixels (for redundancy). These statistics define the linear and quadratic terms of the quadratic unconstrained binary optimization (QUBO) formulation. To make the problem compatible with quantum hardware, the QUBO is downsampled spatially and sparsified to reduce connectivity. A soft linear constraint enforces the selection of a fixed number of features, and the QUBO is then submitted to the quantum annealer. The selected pixels are treated as a compressed representation, which is used to train a decoder for image reconstruction.

Feature selection (FS) aims to mitigate these challenges by identifying a subset of relevant features that contribute most significantly to a target variable. Effective FS reduces input dimensionality, improves model interpretability, lowers computational complexity, and often enhances generalization performance^3^. In medical imaging, FS can also have direct clinical applications, such as minimizing radiation exposure by identifying the most informative measurements for acquisition^4,5^.

Traditional FS methods are categorized into *filter methods*, which evaluate features based on statistical metrics like mutual information (MI), and *embedded methods*, which incorporate FS into model training. In particular, MI quantifies the dependence between variables without assuming linearity, making it a widely used and theoretically justified criterion for supervised FS^6,7^.

Identifying the optimal subset of features from a set of $n$ candidates is combinatorially complex, requiring evaluation of $$\left( {\begin{array}{c}n\\ k\end{array}}\right)$$ possible subsets for $k$ selected features. To manage this intractability, FS can be reformulated as a quadratic unconstrained binary optimization (QUBO) problem, enabling the use of powerful combinatorial optimization techniques.

In our study, we systematically compare a range of feature selection strategies. As a baseline, we include random feature selection, which provides a lower-bound for performance. To leverage the spatial structure inherent in imaging data, we implement a sampling-based method selecting pixels equally spaced throughout the image. For information-theoretic selection, we formulate a MI-based QUBO and solve it using the classical heuristic solver qbsolv. Additionally, we consider *unsupervised* dimensionality reduction via Sparse Principal Component Analysis (SPCA)^8^, which aims to find sparse latent representations of the input data, and *supervised* feature selection using the Lasso^9^, which identifies features predictive of the target through $\ell _1$-regularization. Finally, we evaluate an autoencoder (AE) approach, where a bottleneck neural network is trained to learn a compact representation of the input without supervision. Together, these methods span randomized, statistical, sparsity-based, and learned paradigms, providing a comprehensive benchmark for evaluating quantum annealing-based FS approaches in medical imaging.

Notably, QUBO formulations are well-suited for emerging quantum computing methods, including adiabatic quantum computing (AQC), which leverage quantum phenomena to potentially explore large solution spaces more efficiently than classical approaches^10,11^. Quantum annealing (QA), a practical realization of AQC, solves QUBO problems by evolving a quantum system from an easily prepared ground state towards the ground state of a complex problem Hamiltonian^12–14^. Commercial quantum annealers, such as those developed by D-Wave Systems^15^, offer access to hundreds to thousands of qubits connected in sparse topologies suitable for optimization tasks. Although current quantum devices fall under the category of noisy intermediate-scale quantum (NISQ) hardware^16^ and remain limited in qubit count and precision, they provide an opportunity to explore QUBO-based FS at non-trivial problem scales. While QUBOs can, in principle, also be solved on gate-based quantum computers using the Quantum Approximate Optimization Algorithm (QAOA)^17^, such approaches remain infeasible at present due to limited qubit availability and hardware noise and are not investigated in this manuscript.

Recent work has investigated MI-based QUBO models for FS, executed on simulated and real quantum hardware^18,19^. D-Wave’s tutorial^18^ introduced a basic framework for MI-based FS on toy datasets. Building on this, Muecke et al.^19^ demonstrated MI-QUBO FS for MNIST and synthetic datasets, including image compression tasks. However, due to hardware limitations, these studies were restricted to very small problem sizes ($n\le 34$) on quantum devices. A recent study by Hellstern et al.^20^ also investigates quantum and classical methods for feature selection formulated as a QUBO problem, with a focus on determining the optimal number $k$ of selected features. In contrast, our work assumes a fixed $k$ and investigates how well various methods, including QA, can select informative subsets under this constraint for use in compression and reconstruction tasks. Further efforts have extended QA-based FS to areas such as hyperspectral image classification^21^, RNA sequencing data analysis^22^, recommendation systems^23^, and radiomics feature selection^24^. Beyond FS, QA has also been explored in medical imaging applications including tomographic reconstruction^25–27^, segmentation^28^, and super-resolution tasks^29^.

In this work, we explore how QA can be applied to FS in medical imaging using light-weight datasets. Our focus is on demonstrating FS at larger scales than previously achieved on real quantum hardware, by carefully adapting the QUBO construction to current architectural limitations. Specifically, our experiments are conducted on the MedMNIST benchmark^30^, which comprises standardized $28 \times 28$ pixel images across multiple imaging modalities. We treat each pixel as an individual feature, recognizing that pixels are not optimal descriptors but offering a manageable and standardized starting point for proof-of-concept experiments.

**Fig. 2:**
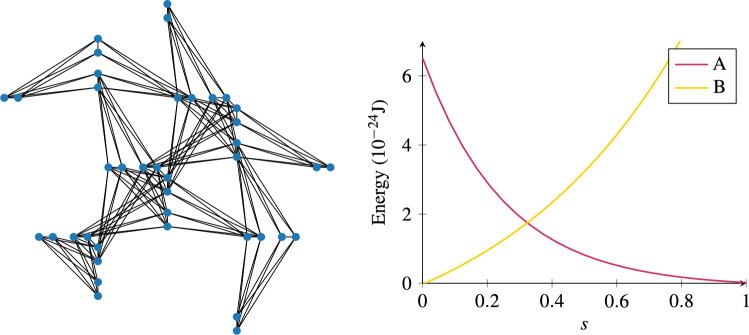
(Left) A part of the Pegasus topology implemented in the quantum annealing D-Wave Advantage_system4.1 architecture, where qubits (blue circles) are connected with couplings (black lines) to a maximum of 15 other qubits. (Right) General example of anneal schedule parameters, where *A*(*s*) and *B*(*s*) scale the transverse field and Ising contributions, respectively. These coefficients are functions of the parameter $s \in [0,1]$, which depends on the physical time *t*. In specific hardware implementations, like the D-Wave Advantage_system4.1, these parameters may take different forms.

Our contributions are threefold: First, we implement MI-based QUBO FS on six light-weight medical imaging datasets and demonstrate feasibility on a classical solver. Second, we introduce hardware-aware adaptations including subsampling, thresholding, and a sparsity-preserving linear Ising penalty to enable embedding of larger QUBO instances on a QA system. Finally, we demonstrate the utility of the selected features by training a convolutional encoder for lossy image compression, illustrating potential applications of FS beyond classification. Although constrained by current hardware limitations, this study provides a proof of concept for applying quantum optimization methods to FS tasks in medical imaging. While this study focuses on a simplified toy problem, the approach could inform potential clinical applications such as radiation dose reduction during image acquisition^4,5^ and improved diagnostic interpretability by identifying key image regions.

## Quantum annealing

We focus on the particular implementation of AQC known as QA, in which the problem to be solved is mapped to the minimization of an Ising Hamiltonian, in order to be embeddable in a quantum annealer. FS is also suitable for this technique since it can be formulated as a QUBO problem, which in turn can easily be mapped to an Ising system. QA operates on the fundamental concept of quantum tunneling and the quantum adiabatic theorem to explore the vast solution space of a problem in order to find the optimal solution of the Ising Hamiltonian, which corresponds to a global minimum of the optimization function. It is important to note that while QA has shown promise in a variety of fields^31–38^, it is not a universal quantum computing approach like gate-based quantum computers. Indeed, future gate-based quantum computers are versatile and capable of performing a wide range of computations, including optimization of QUBOs via algorithms such as the QAOA^17^. In contrast, quantum annealers are specialized devices tailored for optimization using adiabatic quantum evolution.

This is not a limitation for our analysis, as our problem can be cast into a form embeddable in a quantum annealer. The QA device we use in this study is developed by D-Wave Systems, featuring specialized hardware that generates and sustains the necessary quantum states. These devices use superconducting qubits and magnetic fields to create controlled quantum environments where the annealing process takes place. In particular, we shall perform our analysis on the Advantage_system4.1 architecture^15^: this annealer contains 5627 qubits, connected in a Pegasus structure, but only has a total of 40279 couplings between them. This topology is depicted in Fig. 2.

The full Hamiltonian in QA comprises a time-dependent mixture of a known driver Hamiltonian, responsible for inducing quantum fluctuations, and a problem Hamiltonian, which encodes the classical Ising objective to be minimized. The original idea behind QA (and AQC more generally) is to begin in the ground state of the trivial system and adiabatically replace the trivial Hamiltonian with the problem Hamiltonian, while remaining in the ground state throughout. In particular, the mixed Hamiltonian takes the following form:

1 $$\begin{aligned} \mathscr {H} ~=~ \, A(s)\sum _i\sigma _i^x \, + B(s) \, \left( \sum _{ij} J_{ij} \sigma _i^z \sigma _j^z \, + \, \sum _i h_i \sigma _i^z\right) \,, \end{aligned}$$

where *i*,*j* label the qubits, $\sigma _i^z$ are the *z*-spin Pauli matrices, and $\sigma _i^x$ are the transverse field components, while the couplings $h_i$ and $J_{ij}$ between the qubits are set and kept constant. These couplings define the classical cost function and the part of the Hamiltonian multiplied by *B*(*s*). The parameter *s*(*t*) (with *t* being time) is a user-defined control-parameter that can be adjusted, while *A*(*s*) and *B*(*s*) describe the consequent change in the quantum characteristics of the annealer. The network of qubits starts in a global superposition over all possible classical states and, as *s* approaches a value of 1, the system localises into a single classical state once a measurement of $\sigma _i^z$ on all sites has been performed. The anneal schedule increases linearly with time, with $s(0)=0$ and $s(t_f)=1$, where $t_f$ is the total annealing time. At early anneal times ($s \approx 0$), the system is governed by the transverse field *A*(*s*), promoting quantum superposition and tunneling. As *s* approaches 1, the system transitions to the classical problem Hamiltonian. To solve the optimization problem, the problem is encoded into the classical component of the annealing Hamiltonian, specifically, the term multiplied by *B*(*s*) in Eq. (1). This term, referred to as the problem Hamiltonian $H_P$, defines a classical energy landscape in the form of a generalized Ising model:

2 $$\begin{aligned} H_P ~=~ \sum _{ij} \, J_{ij} \, \mu _i \mu _j \,+\, \sum _i \, h_i \, \mu _i \,, \end{aligned}$$

where $\mu _i \in \{-1/2, 1/2\}$ are the eigenvalues of the spin operator $\sigma _i^z$. The coefficients $J_{ij}$ and $h_i$ encode the binary quadratic objective function as Ising couplings and local fields, respectively. If the process remains adiabatic, the final state of the system corresponds to a lowest-energy configuration solving the original problem. Since real-world QA may not always reach the global minimum due to noise and non-adiabatic effects, the process is typically repeated many times to gather a distribution of solutions, from which the best one is selected.

**Fig. 3:**
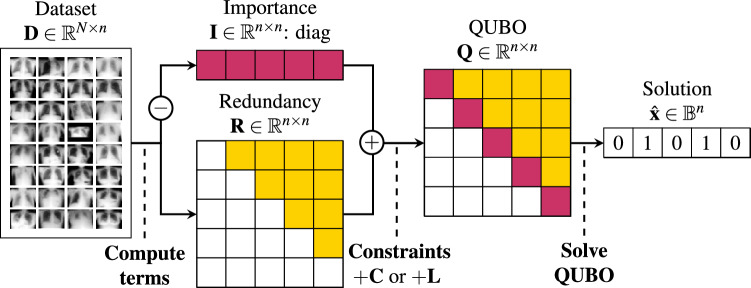
Feature selection pipeline: Images are flattened to compute the importance and redundancy terms, which are combined into the QUBO. The *k* of *n* constraint is enforced via a linear penalty or a quadratic constraint (Fig. 4). Then, the QUBO is solved using classical or quantum solvers.

## Methods

### Mutual information feature selection QUBO

The goal for our FS method is to define an optimization objective tailored to a QUBO and complying with the constraints introduced by the current hardware. Consider an image dataset consisting of square, two-dimensional images with width $W$, paired with their labels. The training dataset can be represented as $D = \{(\bf{X}^i, y^i)\}_{i \in [N]}$, where each $\bf{X}^i \in {\mathbb {R}}^{W \times W}$ is an image, and $y^i \in {\mathbb {N}}$ is its corresponding class label. By flattening the images, the dataset transforms into $D_f = \{(\bf{x}^i, y^i)\}_{i \in [N]}$, where $\bf{x}^i \in {\mathbb {R}}^n$ represents the $n$-dimensional vectorized data, and $y^i$ remains the class label.

While the class label $y^i$ is not used in the subsequent image reconstruction process, it plays a crucial role in feature selection. Specifically, we propose a model-agnostic FS task, where we want to maximize the mutual information (MI) of the features with the class label to determine feature relevance. The selected features are then used the image reconstruction task, independent of $y^i$. In our simplified example, we will treat the image pixels as features and formulate the pixel selection problem as a QUBO model:

3 $$\begin{aligned} \min (f_{Q}(\hat{\bf{x}})) = \hat{\bf{x}}^{T} \bf{Q} \hat{\bf{x}} = \sum _{i=1}^{n} Q_{i,i} \hat{x}_{i} + \sum _{i=1}^{n} \sum _{j>i}^{n} Q_{i,j} \hat{x}_i \hat{x}_j \,. \end{aligned}$$

Here, $\hat{x}_{i}$ indicates whether a feature is selected (1) or not (0), where $\hat{\bf{x}} \in \{0,1\}^n$. The linear terms originate from the binary nature of $\hat{x}_{i}^{2} = \hat{x}_{i}$. In turn, our matrix $\bf{Q} \in {\mathbb {R}}^{n \times n}$ describes our optimization problem. In the subsequent steps, we show how we construct the QUBO matrix from the information contained in the image datasets. Intuitively, feature $x_i$ is more likely to be selected if $Q_{i,i}$ is low. Similarly, we can increase the chances of choosing feature $x_{i}$ and feature $x_{j}$ together if the $Q_{i,j}$ term is small. The diagonal elements $Q_{i,i}$ of the matrix $\bf{Q}$ encode the importance of each feature and are defined as the negative MI between feature $x_i$ and the class label *y*. The off-diagonal elements $Q_{i,j}$ (for $i \ne j$) represent the redundancy between features, calculated as the MI between features $x_i$ and $x_j$. Together, these define a binary quadratic objective function that balances feature relevance and redundancy. This model closely resembles the form of an Ising problem, and in fact one can translate a QUBO into an Ising problem by mapping $x_i \rightarrow \mu _i + \frac{1}{2}$, where $\mu _i$ is defined as in Eq. (2).

In FS, MI is widely used as it captures nonlinear dependencies and does not assume specific distributions^6,7^. We closely follow the MI FS QUBO described in^18,19^. To calculate MI, we estimate the joint and marginal probabilities of the features and labels. For efficient computation with continuous, real-valued features, this requires discretization. We divide each feature dimension into $n_{bins} = 20$ bins using quantiles, assigning each feature value to its corresponding bin. Labels, being discrete, do not require binning. This discretization and probability estimation allows for efficient computation of MI between features and labels. Please refer to^19^ for a detailed explanation of this method. From the discretized dataset $\hat{D} = \{(\bf{b}^{i}, y^i)\}_{i \in [N]}$, the empirical joint and marginal probabilities are defined as follows:

4 $$\begin{aligned} \hat{p}_{X, Y}(b, y)= & \frac{1}{N} \sum _{j=1}^N \mathbbm {1}(b_j = b \wedge y_j = y) \end{aligned}$$

5 $$\begin{aligned} \hat{p}_{X_i, X_j}(b_i, b_j)= & \sum _{y} \sum _{b_{k \ne i,j}} \hat{p}_{X, Y}(b, y) \quad \text {and}\quad \hat{p}_{X_i, Y}(b_i, y) = \sum _{b_{k \ne i}} \hat{p}_{X, Y}(b, y) \end{aligned}$$

6 $$\begin{aligned} \hat{p}_Y(y)= & \sum _{b_k} \hat{p}(b, y) \quad \text {and}\quad \hat{p}_{X_i}(b_i) = \sum _{y} \sum _{b_{k \ne i}} \hat{p}_{X, Y}(b, y) \end{aligned}$$

We initialize our linear QUBO terms with the negative MI of a feature with its class label, which we label as importance:

7 $$\begin{aligned} Q_{i,i} = -I_{i,i} = -I(x_i, y) \approx \sum _{b} \sum _{y} \hat{p}_{X_i, Y}(b, y) \log \frac{\hat{p}_{X_i, Y}(b, y)}{\hat{p}_{X_i}(b) \hat{p}_Y(y)}. \end{aligned}$$

We want to avoid choosing features that share a lot of information. This is achieved by populating the off diagonal term $Q_{i,j}$ with the MI between feature $x_i$ and $x_j$. A high MI value for $Q_{i,j}$ will increase the energy of the solution that selects both the $x_i$ and $x_j$ features decreasing the probability of it being returned by the annealer.

8 $$\begin{aligned} Q_{i,j}= R_{i,j} = R(x_i, x_j) \approx \sum _{b} \sum _{b'} \hat{p}_{X_i, X_j}(b, b') \log \frac{\hat{p}_{X_i, X_j}(b, b')}{\hat{p}_{X_i}(b) \hat{p}_{X_j}(b')}. \end{aligned}$$

**Fig. 4:**
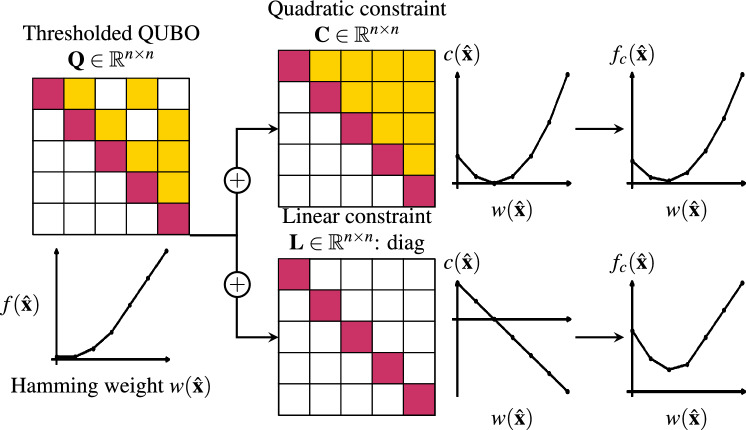
Illustration of the conventional quadratic constraint to enforce selecting *k* of *n* features, which is infeasible due to limited connectivity on the annealer. When dealing with a sparsified QUBO, we propose a linear Ising penalty to enforce the constraint. The QUBO and its constraint are shown, along with a plot of the optimization energy (y-axis) against the Hamming weight (x-axis), indicating how many features are selected $\sum _{i} \hat{x}_{i}$.

Finally, we have to enforce our constraint of choosing *k* of *n* features. To enforce such constraint, an option is to use a quadratic penalty $\alpha (\sum _{i=1}^{n} \hat{x}_{i} - k)^2$, where $\alpha$ must be tuned to weight the constraint appropriately. The quadratic constraint in the form of a QUBO $\bf{C}$ is constructed by $C_{i,i} = 1 - 2k$ and $C_{i,j} = -2n + 2$, resulting in a fully connected problem graph. We construct our FS QUBO problem by additively combining the individual matrices: $\bf{Q} = - \bf{I} + \bf{R} + \alpha \bf{C}$, where $\bf{I}$ is the diagonal importance matrix, $\bf{R}$ is the redundancy matrix filling the quadratic couplings, and $\bf{C}$ enforces the feature count constraint. The full procedure of creating the QUBO is depicted in Fig. 3 and an illustration of the quadratic constraint is shown in Fig. 4.

### Mapping the problem to quantum hardware

In the previous section, we described how to construct a MI-based QUBO model for feature selection. While this formulation can be solved using classical algorithms such as simulated annealing or tabu search, executing it on a quantum annealer presents additional challenges due to the limited connectivity of current hardware. Specifically, although modern quantum annealers such as D-Wave’s Advantage system feature thousands of physical qubits, their underlying Pegasus topology imposes constraints on qubit-to-qubit connectivity, necessitating the use of embedding strategies. These embeddings often require multiple physical qubits to represent a single logical variable, creating an overhead that scales with the problem’s connectivity. To adapt our problem to the hardware, we applied a series of structured reductions aimed at decreasing both the dimensionality and the connectivity of the QUBO graph:

First, we partition the image into non-overlapping $2 \times 2$ neighborhoods and select the pixel with the highest MI with the class label from each block. This reduces the number of features from $28 \times 28 = 784$ to $14 \times 14 = 196$ while preserving local spatial diversity and maximizing information retention. The block size of $2 \times 2$ was chosen as the smallest non-overlapping subsampling unit compatible with the original $28 \times 28$ image size. It offers a trade-off between granularity and QUBO tractability: larger blocks would further reduce dimensionality but risk discarding important local information.

Despite the dimensionality reduction, the resulting QUBO remains densely connected due to the presence of pairwise redundancy terms between features. To address this, we apply a thresholding strategy that discards weak quadratic couplings, defined as those with low MI between feature pairs. Specifically, we retain the top 2000 largest $Q_{ij}$ terms in absolute value. This threshold level was empirically determined to balance two competing objectives: maintaining enough pairwise structure to preserve problem fidelity, while reducing graph density to ensure embeddability within the limited connectivity of the hardware. The resulting sparsified QUBO retains essential interactions while substantially reducing embedding overhead.

Rather than using a fully connected quadratic penalty term to enforce the cardinality constraint $\sum _i \hat{x}_i = k$, which would reintroduce dense connectivity, we use a sparsity-preserving linear penalty approach. This introduces an adjustable weight $\alpha _l$ on the diagonal of the QUBO, as described in the following section, and allows for consistent enforcement of the feature count constraint with reduced connectivity overhead. Together, these steps result in a QUBO instance that can be embedded in the D-Wave Advantage_system4.1 architecture. The final annealing parameters, including annealing time, number of reads, number of physical qubits and average chain length are described in the experimental section.

### Linear Ising penalties

To limit the connectivity of the QUBO model while still enforcing the constraint, we propose using a linear penalty term, $\left| \sum _{i=1}^{n} \hat{x}_{i} - k \right|$, similar to the approach presented in^39,40^. This linear penalty, denoted as $\bf{L}$, introduces an offset $\alpha _{l}$ on the diagonal of the QUBO matrix. The parameter $\alpha _{l}$ is tuned to achieve the desired Hamming weight of the solution vector $\hat{\bf{x}}$, where the Hamming weight corresponds to the number of selected features in the solution.

For each dataset, we tune the linear penalty coefficient $\alpha _l$ through a deterministic search procedure to ensure that the QUBO solution satisfies the hard constraint $\sum _i x_i = k$. Starting from a small initial value, $\alpha _l$ is incrementally increased until the returned solution has exactly *k* selected features. The resulting values are dataset-specific and remain fixed for all subsequent experiments.

When constructing the QUBO we substitute $\bf{C}$ for $\bf{L}$: $\bf{Q} = - \bf{I} + \bf{R} + \alpha _{l} \bf{L}$. An overview and visual comparison of the constraint creation process for the QUBO is provided in Fig. 4. While the linear penalty constraint may be less effective for certain problem instances, our experiments show it consistently enforces the desired behavior.

### Reconstruction decoder

Our experiment focuses on reconstructing images from the selected subset of pixels. Once the features are extracted for each dataset, we train a convolutional decoder to reconstruct the original image from the selected pixels. An illustration of the procedure in displayed in Fig. 1. The reconstruction network consists of a linear layer followed by two two-dimensional transposed convolutional layers, each followed by a ReLU activation function and then a sigmoid activation function.

### Datasets

For our experiments, we used the MedMNIST dataset^30^, which contains 18 standardized datasets used for biomedical image classification. The collections compromise 12 two-dimensional and 6 three-dimensional datasets. The collection contains data scales from a few hundred to 100,000 and binary and multi-class classification tasks. In particular, the dataset collection should facilitate light-weight machine learning research in medical imaging without directly facing clinical challenges. Due to the size restrictions of our QA device, we only analyze the two-dimensional grayscale image datasets. We note that ChestMNIST, OCTMNIST, PneumoniaMNIST and BreastMNIST consist of images that are semi-registered. This plays an important role when discussing pixels as feature descriptors. The datasets analyzed are summarized in Table 1.

**Table 1:** MedMNIST v2 2D datasets used for the feature selection experiments in this manuscript. Table adapted from^30^.

Dataset $(28 \times 28)$	Modality	Tasks (# Classes)	# Samples	Train/Test
ChestMNIST	Chest X-ray	Binary (2)	112,120	78,468/22,433
OCTMNIST	Retinal OCT	Multi (4)	109,309	97,477/1,000
PneumoniaMNIST	Chest X-ray	Binary (2)	5,856	4,708/624
BreastMNIST	Breast ultrasound	Binary (2)	780	546/156
TissueMNIST	Microscope	Multi (8)	236,386	165,466/47,280
OrganAMNIST	Abdominal CT	Multi (11)	58,830	34,561/17,778

**Fig. 5:**
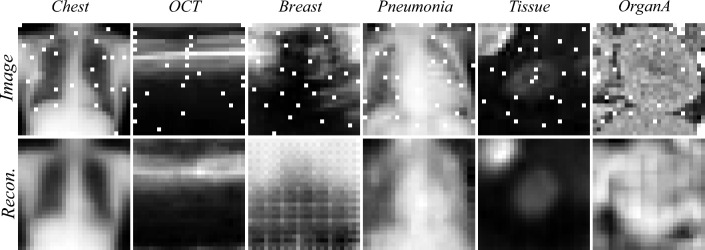
Visual comparison of images from the test set, overlayed with the selected pixels (top row) and the decoder reconstructed images from the selected pixels.

## Results

### Reconstruction experiment

We compare different FS methods for selecting subsets of features in our reconstruction experiments. The FS methods evaluated include: (1) a random sampling approach that selects *k* features at random, (2) a subsampled approach that evenly spreads the pixels across the image in a grid-like fashion, (3) supervised feature selection using Lasso regression, where features are selected based on their regression coefficients with respect to the target labels, (4) the full QUBO formulation solved using the classical tabu-search solver qbsolv^41^, (5) our proposed QA method, which solves a sparsified QUBO adapted for hardware constraints, (6) unsupervised feature compression using SPCA, which generates a sparse representation of the data without supervision, and (7) a learned feature extractor, specifically an AE with a latent dimension of $k=25$, whose encoder resembles the presented decoder architecture.

Beyond these approaches, numerous FS techniques exist, including random forest-based selection^42^, simulated annealing-based selection^43^ or exact solvers like Gurobi^44^. While these were not explicitly compared in our experiments, they represent alternative strategies for feature selection that may be explored in future work.

We trained a convolutional encoder, using feature sets of size $k = 25$, that takes the selected pixels as input to reconstruct the original image. The encoder was trained using the Adam optimizer with a learning rate of 0.001 for 20 epochs, minimizing mean squared error (MSE) loss. Reconstruction performance was validated first on the MNIST dataset, showing results consistent with those reported by Muecke et al.^19^. The experiments were then extended to the MedMNIST dataset. Each selection and training process was repeated five times to calculate the mean and standard deviation. The reconstruction results, expressed in terms of MSE on the test set, are summarized in Table 2. Visual examples of reconstructed images for each dataset using QA-selected pixels are shown in Fig. 5.

### Simulation and hardware experiments

In the classical simulation setup, we performed FS on the full QUBO of size $784 \times 784$, incorporating a quadratic constraint. The QUBO was solved using the qbsolv algorithm, which partitions the problem into smaller subproblems. From the generated sampleset, the solution with the lowest energy was selected. The runtime of qbsolv on the full size QUBO was $5.6 \pm 0.56 \, \textrm{s}$.

To validate the solutions on real quantum hardware, we executed the experiments on the D-Wave Advantage_system4.1 through Leap^TM^ using the associated Ocean Python API^45^. As discussed above, we used a series of steps to reduce the size and connectivity of the QUBO model. The original QUBO was defined on a $28 \times 28$ pixel grid, resulting in a size of $784 \times 784$. To downscale this, we used a subsampling strategy, selecting the pixel with the highest MI from each $2 \times 2$ neighborhood in the image. This reduced the QUBO size to $196 \times 196$. Despite this reduction, the connectivity of the QUBO still exceeded the hardware’s constraints. To address this, we applied a thresholding technique that removed weaker quadratic couplings, retaining 2000 couplings in the final problem representation. This sparsification step aligned with an observed average chain length of $4.51 \pm 0.36$ physical qubits per logical variable and used around $883.17 \pm 82.80$ qubits on the QA hardware. The average chain length and qubit count was calculated as the mean over the six datasets with five idependent runs per sampling.

Minor embeddings were generated dynamically using the EmbeddingComposite from D-Wave’s Ocean SDK, employing heuristic algorithms to map logical variables to the Pegasus P16 topology. Chain strengths were automatically selected by the embedding composite, proportional to the problem QUBO coefficients, to maintain intra-chain consistency without manually tuning. Chain break resolution was handled via majority vote, where the most common qubit value within a chain determines the logical variable’s assignment.

The *k* of *n* constraint was enforced using a sparsity-preserving linear penalty, ensuring compatibility with the hardware while preserving the structure of the problem. The subsampled, thresholded, and linear Ising-constraint-enforced QUBO was mapped to the annealing hardware, with the annealing time set to $t_f = 20 \, \mu s$. We performed 1000 reads to form a sampleset and selected the solution with the lowest energy observed. The runtime on the quantum annealer was $260.84 \pm 12.64 \, \textrm{ms}$. Overhead times, such as queueing the problem to the quantum annealer and preparing the QUBO, were not included in the reported runtime, as these are shared with the qbsolv workflow.

**Table 2:** Evaluation of the image compression task using selected pixels and learned features, measured via test set mean square error. Methods compared include random pixel selection, sampled pixel selection (uniform spacing over the image grid), supervised Lasso selection, solving the full QUBO with qbsolv, solving a sparsified QUBO with quantum annealing (QA), and compression using an autoencoder (AE) and sparse principal component analysis (SPCA). Results are reported as mean ± standard deviation over five independent runs. For QA experiments, sparsified QUBOs were used to match current hardware embedding constraints, while all classical methods, including qbsolv, operated on the full feature space.

Dataset	Random $\times 10^{-3}$	Sampled $\times 10^{-3}$	Lasso $\times 10^{-3}$	qbsolv $\times 10^{-3}$	QA $\times 10^{-3}$	AE $\times 10^{-3}$	SPCA $\times 10^{-3}$
ChestMNIST	$6.7 \pm 0.6$	$6.0 \pm 0.0$	$9.3 \pm 0.1$	$5.3 \pm 0.2$	$5.5 \pm 0.2$	$2.3 \pm 0.0$	$3.0 \pm 0.0$
OCTMNIST	$6.7 \pm 1.8$	$5.9 \pm 0.1$	$17.3 \pm 0.1$	$4.7 \pm 0.1$	$5.3 \pm 0.5$	$1.4 \pm 0.1$	$2.6 \pm 0.0$
PneumoniaMNIST	$6.9 \pm 0.5$	$5.9 \pm 0.4$	$7.3 \pm 0.2$	$5.5 \pm 0.1$	$5.3 \pm 0.2$	$3.1 \pm 0.1$	$2.9 \pm 0.0$
BreastMNIST	$22.3 \pm 3.5$	$23.5 \pm 1.3$	$22.1 \pm 2.6$	$21.8 \pm 3.0$	$21.2 \pm 2.6$	$14.8 \pm 1.7$	$4.5 \pm 0.0$
TissueMNIST	$3.6 \pm 0.2$	$3.1 \pm 0.0$	$4.4 \pm 0.0$	$3.3 \pm 0.1$	$3.0 \pm 0.1$	$1.6 \pm 0.0$	$1.9 \pm 0.0$
OrganAMNIST	$39.4 \pm 0.9$	$37.2 \pm 0.3$	$40.1 \pm 0.1$	$40.3 \pm 0.4$	$37.4 \pm 0.5$	$24.7 \pm 0.2$	$25.2 \pm 0.0$

## Discussion

In this work, we presented a method to encode a FS problem that can be implemented on commercially available quantum computing hardware. Our approach focuses on selecting the *k* most important features, as measured by MI, from six light-weight medical image datasets. We evaluated the selected features by training a convolutional reconstruction decoder and measuring the MSE of reconstructed samples compared to ground truth test set images.

To address the limitations of quantum hardware connectivity, we enforced a linear penalty to reduce the connectivity of the problem graph. This allowed us to generate a subsampled, thresholded QUBO formulation, that reduces the connectivity and computational complexity of the problem. Despite these simplifications, the solutions obtained on the quantum hardware were quantitatively (see Table 2) and qualitatively (see Fig. 5) comparable to those derived from a simulated solver operating on the complete problem description. This demonstrates that our method effectively balances the trade-off between hardware limitations and solution quality. The performance of our quantum-based FS approach was comparable to that achieved using a classical solver. However, we note that the approximated QUBO may yield suboptimal global solutions due to discarded weak interactions, and future hardware developments or hybrid embedding strategies may alleviate this.

Our experiments showed that the QUBO-based FS method identified plausible features for training the reconstruction encoder. However, the effectiveness of FS was dataset-dependent. For medical imaging datasets with relatively aligned images, such as ChestMNIST, PneumoniaMNIST, BreastMNIST, and OCTMNIST, the method performed well, resulting in meaningful feature subsets that supported accurate reconstructions. In contrast, for datasets where the image content was misaligned or highly heterogeneous, such as cell images, or multi-organ images, the selected features did not outperform simple subsampling. This is consistent with existing knowledge that localized pixel features are suboptimal for these tasks, as they fail to capture global spatial or contextual information.

Beyond evaluating the QUBO-based FS method, we systematically compared it against a range of classical and learning-based FS strategies to establish a broader performance baseline. Random pixel selection served as a lower-bound baseline, providing a reference for performance without structured feature selection. Subsampling, based on uniformly spaced pixel selection, acted as a natural baseline for image tasks, maintaining spatial coverage while ignoring label information. Lasso regression, despite its strong performance in tabular settings, underperformed in our experiments, often selecting spatially clustered pixels without considering redundancy, leading to poor reconstruction quality. In contrast, qbsolv-based solutions consistently outperformed random and subsampling approaches across several datasets, highlighting the value of structured MI-based optimization even with heuristic solvers. Although global solvers such as Gurobi^44^ are not incorporated in the current study due to the computational complexity of solving the complete QUBOs with 784 binary variables, they remain an important benchmark for future work.

SPCA and AE based feature compression methods achieved the lowest reconstruction errors across all datasets. However, as noted by Hellstern et al.^20^, such unsupervised methods generate new transformed features rather than selecting existing ones, necessitating access to the full set of original features at deployment. This limitation highlights why neural networks, which excel at learning hierarchical and spatially invariant representations, are particularly effective for such tasks. Our QUBO-based method, by contrast, identifies explicit subsets of raw pixels, offering direct applicability to tasks such as optimizing imaging acquisition protocols, reducing radiation dose, and enhancing diagnostic interpretability. It is important to note that the objective of this study was not to propose a new feature extractor but rather to select a subset of features from a large set that adequately describes the data distribution. Learned feature descriptors, such as those produced by neural networks, offer unparalleled performance due to their ability to generalize across large datasets, but they often lack interpretability. In contrast, our QUBO-based approach provides interpretable FS grounded in statistical measures such as MI and redundancy, offering insights into the most relevant features for specific tasks.

Integrating learned feature representations with interpretable FS methods could present a powerful hybrid approach. For instance, features extracted from foundation models or other pre-trained neural networks could be used as inputs to our QUBO-based framework, combining the strengths of deep learning with the interpretability and sparsity benefits of quantum-inspired FS. This would enable both effective and interpretable solutions, bridging the gap between data-driven learning and human-comprehensible FS. Additionally, future work could investigate the incorporation of more advanced quantum algorithms or optimizing hardware configurations to further improve scalability and performance.

## Conclusion

In this work, we provided an introduction to using quantum annealers for feature selection in an image compression task. We presented a method to perform feature selection on currently available quantum annealing hardware and applied it on light-weight medical imaging datasets. The method outperforms other simple feature selection techniques, but cannot compete against trainable deep learning based feature extractors. By leveraging an adapted QUBO formulation with thresholding, subsampling and hardware optimized constraints, we demonstrated how quantum hardware can be used effectively despite its current limitations. This work highlights potentials of quantum feature selection as a foundation for future explorations in interpretable and scalable feature selection methodologies, possibly combining deep learning based feature extractors and quantum based feature selection.

## Data Availability

The datasets analyzed during this work are publicly available and can be found at: https://medmnist.com/.
